# Comparison of Self-Collected Versus Clinician-Collected Human Papillomavirus (HPV) Sampling for Cervical Cancer Screening: A Pilot Study in the United Arab Emirates

**DOI:** 10.7759/cureus.89250

**Published:** 2025-08-02

**Authors:** Shalini Fernandes, Nabilah Mashharawi, Ayisha Ejaz Bhutta, Rupa Murthy Varghese, Aydah Belal Al Ali, Eiman Saeed Al Zahmi, Muna Abdul Razzaq Tahlak, Nighat Aftab

**Affiliations:** 1 Department of Obstetrics and Gynecology, Latifa Women and Children Hospital, Dubai, ARE; 2 Department of Obstetrics and Gynecology, Mohammed Bin Rashid University of Medicine and Health Sciences, Dubai, ARE; 3 Department of Pathology and Laboratory Medicine, Latifa Women and Children Hospital, Dubai, ARE

**Keywords:** cancer screening, cervical cancer, clinician-sampling, human papillomavirus, self-sampling

## Abstract

Background: Cervical cancer is one of the leading causes of cancer deaths in women in the United Arab Emirates (UAE), with most deaths attributed to late detection. Most of the cervical cancer cases are linked to infection with sexually transmitted ‘high-risk’ types of human papillomavirus (HPV). Numerous studies have established the superiority of HPV detection in cervical samples compared to cervical cytology for the primary screening of cervical cancer. Self-collection of samples is found to be very acceptable and favored by the majority of women globally, making it a prospective way to increase women's participation in routine cervical cancer screening.

Aims: This study was done to compare the results of self-collected HPV samples versus clinician-collected samples by using the same device (Qvintip, Aprovix, Stockholm, Sweden). Also, to find out the patient-reported acceptability and preference for the sampling technique, a feedback form was used.

Methods and materials: One hundred eligible women were selected at random (convenience sampling) during their visit to the Gynecology Department of Latifa Hospital, Dubai, over six months. Self-collected samples were collected by the women in the hospital, followed by clinician sampling using the Qvintip sampling device, and analyzed using the Aptima HPV assay, Panther System (Hologic, Inc, Massachusetts, USA). All participants were given a short questionnaire after the sampling procedure regarding the acceptability and preference of the sampling technique.Descriptive statistics like mean, median, and standard deviation were used to report the participant characteristics. Cross-tabulation analysis was done to calculate the p-value and kappa value (measure of agreement) between the two sampling methods. Inferential statistical analyses were conducted to explore associations between participant demographics and test results, as well as preferences for sample collection methods.

Results: The findings of this study indicate a high level of agreement between self-collected and clinician-collected samples, as demonstrated by a statistically significant kappa value of 0.889 and a p-value of < 0.001. The prevalence of high-risk HPV was 9% in self-collected samples and 11% in clinician-collected samples, demonstrating consistency in detection rates between the two methods. The feedback from participants underscores the acceptability of self-sampling, with 95% finding it easy to perform, 91% reporting comfort, and 84% reporting no pain during the procedure. Also, 50% of participants expressed a preference for self-sampling over clinician collection. The chi-square test revealed no statistically significant association (χ²(2) = 0.012, *p* = 0.994), suggesting that preference is similar regardless of nationality. Inferential statistical analyses revealed no significant differences between participant demographics (age, BMI, and parity) and HPV detection rate.

Conclusion: This study highlights the feasibility, acceptability, and effectiveness of HPV self-sampling as a primary screening for cervical cancer within a multicultural population in the UAE. Our findings indicate a high level of agreement between self-collected and clinician-collected HPV samples. More than 90% of women were happy with the self-sampling technique, indicating its feasibility as a culturally acceptable and reliable alternative for cervical cancer screening in the UAE.

## Introduction

Cervical cancer ranks as the fourth most prevalent cancer among women globally, with approximately 660,000 new cases and around 350,000 deaths in 2022 [1]. According to 2020 estimates, each year, approximately 123 new cervical cancer cases are diagnosed in the UAE, and an estimated 59 women die from it [2]. Cervical cancer is the fifth leading female cancer overall and the third most common female cancer in women aged 15 to 44 years in the UAE [2]. About 99% of all cervical cancer cases are linked to infection with sexually transmitted ‘high-risk’ types of human papillomavirus (HPV) [1,3]. Cervical cancer is often treatable when diagnosed early and managed effectively. Effective screening through either cytology (Pap smear) or HPV testing and early treatment for precancerous lesions are important for the secondary prevention of cervical cancer [1].

In recent years, HPV detection in cervical samples has been demonstrated to be more effective than cervical cytology for the primary screening of cervical cancer. Self-sampling involves women collecting their own sample for cervical screening using a vaginal swab [3]. Self-collection of samples is found to be very acceptable and favored by the majority of women globally, making it a prospective way to increase women's participation in routine cervical cancer screening [4]. It has substantial advantages over traditional sampling in terms of cost, coverage, and patient convenience [3]. But the self-sampling for HPV is currently not practiced in the UAE. In the UAE, cultural norms around modesty and privacy may deter women from clinician-based cervical screening. Self-collection offers a culturally acceptable alternative, promoting autonomy and potentially improving screening uptake. We find it of the utmost necessity to explore the possibility of using self-sampling techniques in HPV detection in the UAE, which will help in screening the women who are hesitant to come to the hospital.

This study was conducted to compare the results of self-collected samples by using a self-sampling device (Qvintip, Aprovix, Stockholm, Sweden) versus clinician-collected samples by using the same device and using the Aptima HPV assay, Panther System (Hologic, Inc., Massachusetts, USA) of E6/E7 mRNA for 14 high-risk types as the primary outcome. The secondary outcome was patient-reported acceptability and future preference for the collection technique. This study highlights the feasibility of self-sampling in the UAE context and supports its integration into local screening programs to overcome cultural barriers and enhance early detection efforts.

## Materials and methods

Study design and setting

This was a cross-sectional comparative study conducted at the Gynecology Clinic of Latifa Hospital, Dubai, over a nine-month period, from July 1, 2022 to March 31, 2023.

Participants and Sample Size

A total of 100 sexually active women aged 25 to 65 years, eligible for cervical cancer screening, were recruited during their routine Gynecology clinic visits.

Inclusion and Exclusion Criteria

Women were included if they were sexually active, aged between 25 and 65 years, non-pregnant, and due for cervical cancer screening. Women previously treated for cervical intraepithelial neoplasia (CIN), such as through cone biopsy or large loop excision of transformation zone (LLETZ), and attending follow-up or colposcopy appointments were also eligible. Exclusion criteria included active menstrual bleeding at the time of sampling, unwillingness to self-sample, or a history of total hysterectomy.

Methodology

Participants received a written information leaflet and a pictorial guide explaining the self-sampling process and provided written informed consent prior to participation. Each woman collected a vaginal sample using the Qvintip device in a private room at the hospital, followed by clinician-collected sampling using the same device. Clinicians were trained on the sampling procedure before the study commenced. All samples were labeled, anonymized, and sent for HPV testing in the laboratory using the Aptima HPV assay. After sample collection, participants completed a structured questionnaire evaluating the acceptability of and preference for each sampling method, including factors such as ease, comfort, pain, embarrassment, confidence, and overall preference (Appendix 1).

HPV DNA Analysis in the Laboratory

Collected samples were analyzed using the Aptima HPV assay on the Panther System, which detects E6/E7 viral mRNA from 14 high-risk HPV types (16, 18, 31, 33, 35, 39, 45, 51, 52, 56, 58, 59, 66, and 68) without distinguishing between them. Samples that tested positive were further analyzed using the Aptima HPV 16/18/45 genotype assay to specifically identify the presence of HPV types 16, 18, and 45 [5].

Data collection and statistical analysis

Participant data and HPV results from the laboratory were recorded in a Microsoft Excel sheet (Microsoft Corporation, Washington, USA) and analyzed using Statistical Packages for Social Sciences (SPSS) Statistics for Windows, version 26.0 (released 2019, IBM Corp., Armonk, NY). Descriptive statistics such as mean, median, and standard deviation were used to summarize participant characteristics, and frequencies and percentages summarized questionnaire responses. Agreement between the two sampling methods was assessed using cross-tabulation and kappa statistics; a p-value ≤ 0.05 was considered statistically significant.

Normality of demographic data was assessed using the Shapiro-Wilk test, which is an inferential test used to determine whether sample data deviate significantly from a normal distribution. Inferential statistical analyses were conducted to assess associations between participant demographics and test results in both groups. Age, BMI, and parity were analyzed using independent samples t-tests or Mann-Whitney U tests, as appropriate. A chi-square test was used to evaluate the association between nationality (UAE versus non-UAE) and sample collection preference (self-collection, clinician-collection, or not sure).

Ethical Considerations

The study was conducted in accordance with the principles of the Declaration of Helsinki. It was approved by the Dubai Scientific Research Ethics Committee, Dubai (DSREC-04/2022_03, dated June 16, 2022). Written informed consent was obtained from all participants, and confidentiality of participant information was strictly maintained with access restricted to authorized research personnel.

## Results

Participant characteristics 

A total of 100 women were recruited for this study. The median age at enrollment was 41 years (range: 25-58 years), and the median BMI of the participants was 29 kg/m² (range: 21-44 kg/m²). Most participants were multiparous, with parity ranging from 0 to 13 (Table 1).

**Table 1:** Descriptive statistics (age, BMI, and parity) of all 100 study participants

Characteristic	Mean	Median	Standard deviation	Minimum-Maximum
Age (years)	40.31	41.0	7.94	25-58
BMI (kg/m²)	30.004	29.00	5.2615	21-44
Parity	3.27	3.00	2.453	0-13

**Figure 1:**
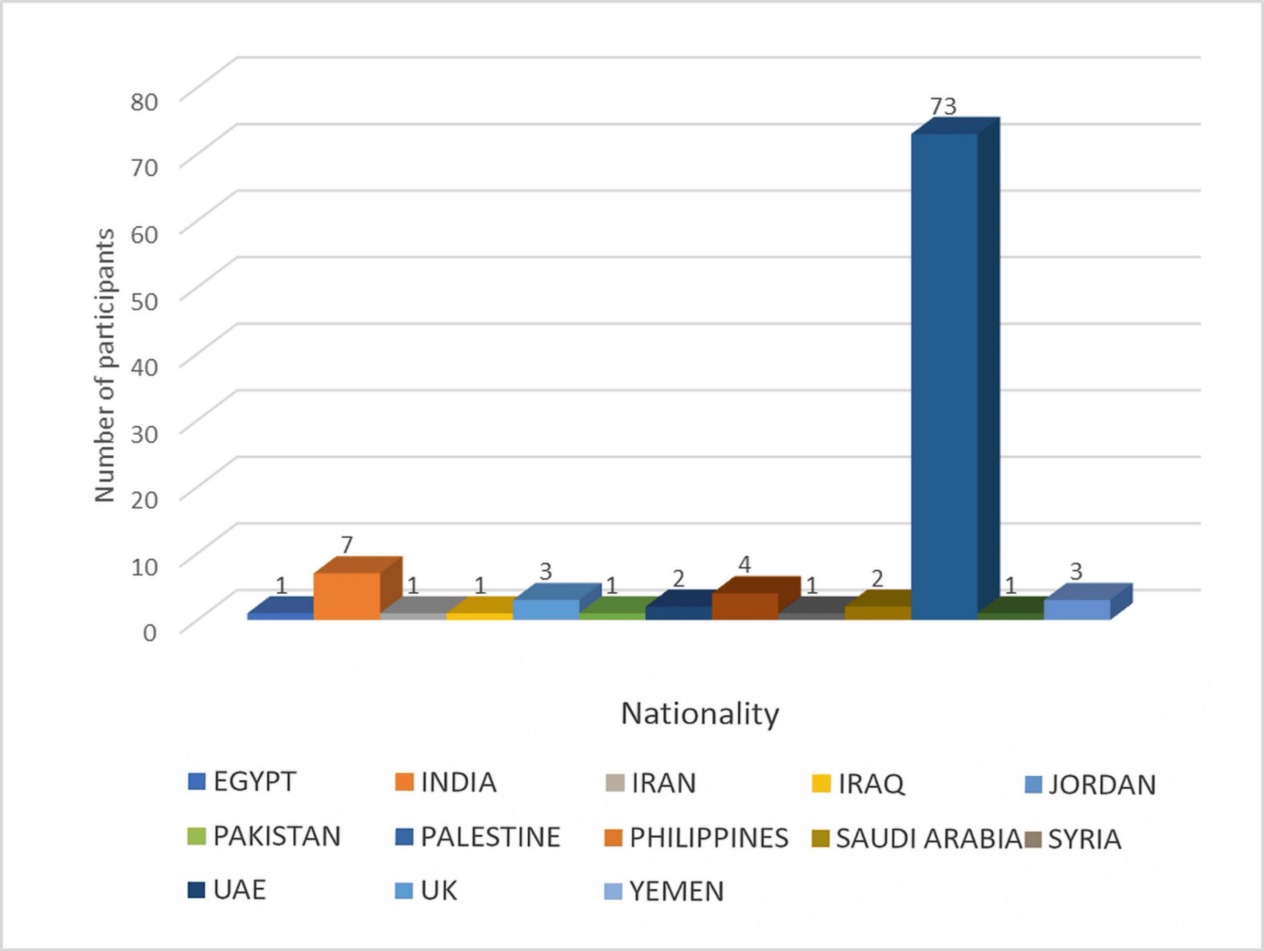
Nationalities of all 100 study participants This figure depicts that 73 (73%) were citizens of the UAE, and the remaining 27 (27%) participants were expatriates. The expatriates subgroup included individuals from India, Pakistan, the Philippines, and various other countries. Numbers = percentage, since the total study participants are 100.

Of the study population, 73 (73%) were citizens of the UAE, reflecting the local demographic, as the study was conducted in a government hospital. The remaining 27 (27%) participants were expatriates, highlighting the multicultural nature of the UAE. This subgroup included individuals from India, Pakistan, the Philippines, and various Gulf Cooperation Council (GCC) countries (Figure 1).​​​​​

Normality of age, BMI, and parity was assessed using the Shapiro-Wilk test. Age was normally distributed (p = 0.454), whereas BMI (p = 0.001) and parity (p < 0.001) showed significant deviations from normality. Accordingly, non-parametric tests were used for variables that violated the normality assumption to calculate the association between test results and demographics (Table 2).

**Table 2:** Shapiro-Wilk Test for normality of age, BMI, and parity of 100 study participants Shapiro-Wilk test for normality: p-values < 0.05 indicate a significant deviation from normality, suggesting the variable is not normally distributed. Age was normally distributed, p-value 0.454. BMI and parity deviated significantly from normality; p-values of 0.001 (**) and p < 0.001 (***) denote statistically significant non-normality.

Variable	Shapiro-Wilk p-value	Normality Conclusion
Age	0.454	Normal
BMI	0.001 (**)	Not normal
Parity	<0.001 (***)	Not normal

Test results 

**Figure 2:**
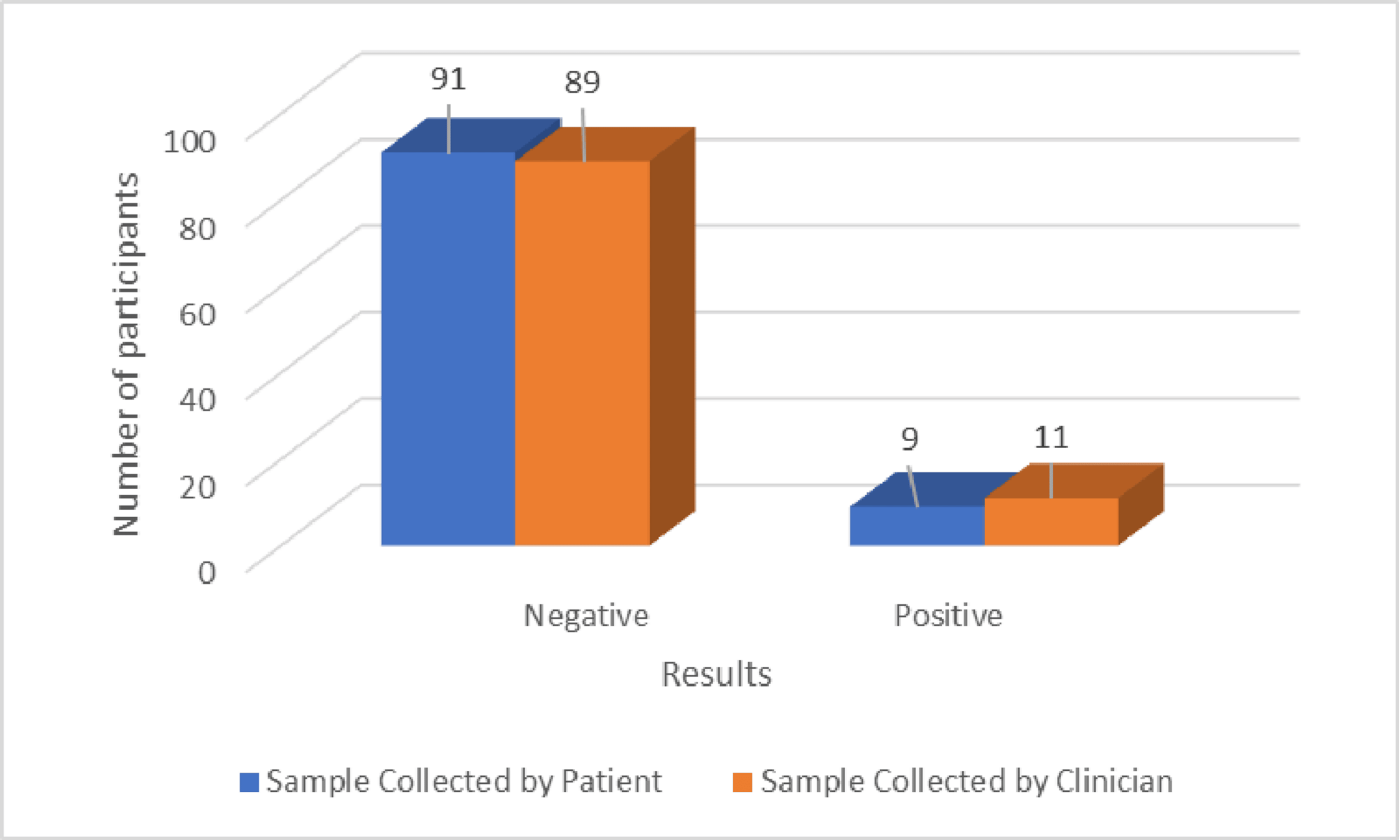
Results of patient-collected and clinician-collected HPV samples Number of participants = percentages, since the total number of participants is 100. HPV: human papillomavirus.

The primary objective of the study was to evaluate and compare the performance of two HPV sample collection methods: self-sampling and clinician sampling. A total of 100 samples were collected by patients through self-sampling, and 100 samples were collected by clinicians. Among the self-collected samples, 91 (91%) tested negative for high-risk HPV, while nine (9%) tested positive. In comparison, 89 (89%) of the clinician-collected samples were negative, and 11 (11%) were positive for high-risk HPV (Figure 2).

Cross-tabulation analysis revealed that 89 (89%) samples were negative across both collection methods (self-sampling and clinician sampling). Only two (2%) samples that tested positive in clinician-collected samples were negative in patient-collected samples, but nine (9%) were positive in both methods. The comparison of results between the two collection methods showed a strong correlation, with a kappa value of 0.889 (indicating substantial agreement) and a statistically significant p-value of <0.001 (Table 3).

**Table 3:** Cross-tabulation of HPV test results between clinician-collected and self-collected samples Values are presented as n (%), where n represents the number of samples. Kappa coefficient of 0.889 indicates agreement between clinician-collected and patient-collected HPV samples and a p-value of < 0.001 (***) indicates strong agreement beyond chance. HPV: human papillomavirus.

		Sample collected by patients n (%)		Kappa value	p-value
Sample collected by clinician n (%)	Results	Negative	Positive	0.889	<0.001***
	Negative	89 (89.0%)	0 (0.0%)		
	Positive	2 (2.0%)	9 (9.0%)		

Association between participant characteristics and test outcomes

Inferential statistical analyses were conducted to explore associations between participant demographics and test results.

Clinician-Collected Samples

No significant differences in demographic or reproductive characteristics were found between participants with positive and negative clinician-collected samples. Participants with negative clinician-collected results had a mean age of 40.31 ± 7.80 years, while those with positive results had a mean age of 40.09 ± 9.74 years. An independent samples t-test showed no significant difference between the groups (t(98) = -0.087, p = 0.931). Median BMI was slightly lower in the positive group, but the difference was not statistically significant (U = 460.5, Z = -0.320, p = 0.749). Parity showed more variability in the negative group; however, the difference between positive and negative groups was not significant (U = 442.0, Z = -0.473, p = 0.636) (Table 4).

**Table 4:** Summary of inferential statistical comparisons between clinician-collected and self-sampling groups A summary of the inferential statistical analyses comparing the age, BMI, parity, and nationality across HPV test result groups. No statistically significant differences were found for any of the variables, with all p-values > 0.05. Effect sizes, where applicable, were negligible, indicating that demographic and clinical characteristics were comparable between participants who tested positive and those who tested negative for high-risk HPV. t: t-test statistic; U: Mann-Whitney U statistic; NSD: no significant difference; HPV: human papillomavirus. p-values are shown directly.

Comparison group	Variable	Statistical test	Test statistic	p-value	Result
Clinician-collected (positive versus negative)	Age	Independent samples t-test	t = -0.087	0.931	NSD
	BMI	Mann-Whitney U test	U = 460.5	0.749	NSD
	Parity	Mann-Whitney U test	U = 442.0	0.636	NSD
Self-collected (positive versus negative)	Age	Independent samples t-test	t ≈ 0.37	> 0.05	NSD
	BMI	Mann-Whitney U test	U = 462.5	0.770	NSD
	Parity	Mann-Whitney U test	U = 440.5	0.630	NSD

Self-Collected Samples

No significant differences in demographic or reproductive characteristics were found between participants with positive and negative self-collected samples. The mean age of participants with negative results was 40.40 ± 7.73 years, while the positive group had a mean age of 39.22 ± 10.67 years. The difference was not significant (t (98) = 0.37, p > 0.05). No significant difference in BMI between groups was observed using the Mann-Whitney U test (U = 462.5, Z = -0.29, p = 0.77). Although the parity values were more spread in the negative group. The Mann-Whitney U test again showed no significant difference between the two groups (U = 440.5, Z = -0.48, p = 0.63) (Table 4).

Secondary objectives

The secondary objective of the study was to assess the acceptability and future preference for the sampling techniques by evaluating variables such as ease, pain, comfort, embarrassment, confidence, and overall preference. It should be noted that not all participants completed the feedback questionnaire in full. All 100 participants provided feedback on the ease of self-sampling. Among them, 58 (58%) rated the process as "very easy," 37 (37%) as "easy," three (3%) as "difficult," and two (2%) as "very difficult" (Table 5).

**Table 5:** Frequency distribution of scores for easiness, comfortability, degree of pain, degree of embarrassment, and confidence in collecting self-sampling Values are presented as n (%), where n represents the absolute frequency.

Dimension	Number of respondents (n (%)	Response Category	n (%)
Easiness	100 (100%)	Very difficult	2 (2%)
		Difficult	3 (3%)
		Easy	37 (37%)
		Very easy	58 (58%)
		Missing data	0%
Comfortability	98 (98%)	Very uncomfortable	1 (1.02%)
		Somewhat uncomfortable	6 (6.12%)
		Comfortable	25 (25.5%)
		Very comfortable	66 (67.34%)
		Missing data	2%
Pain	98 (98%)	Felt pain	14 (14.2%)
		No pain	84 (85.7%)
		Missing data	2%
Embarrassment	97 (97%)	Embarrassed	14 (14.4%)
		Not-embarrassed	83 (85.5%)
		Missing data	3%
Confidence	97 (97%)	Confident	63 (64.9%)
		Not-confident	34 (35.0%)
		Missing data	3%

A total of 98 participants evaluated comfort during self-sampling. Out of these, 66 (67.3%) reported feeling "very comfortable," 25 (25.5%) felt "comfortable," six (6.1%) indicated they were "somewhat uncomfortable," and one (1.02%) felt "very uncomfortable" (Table 5). When asked about pain, 98 participants responded. A vast majority, 84 (85.7%), experienced no pain during the procedure, while 14 (14.2%) reported experiencing pain (Table 5). Of the 97 participants who answered questions regarding embarrassment, 83 (85.5%) reported no feelings of embarrassment, whereas 14 (14.4%) admitted to feeling embarrassed. Additionally, three participants did not provide an answer (Table 5).

**Figure 3:**
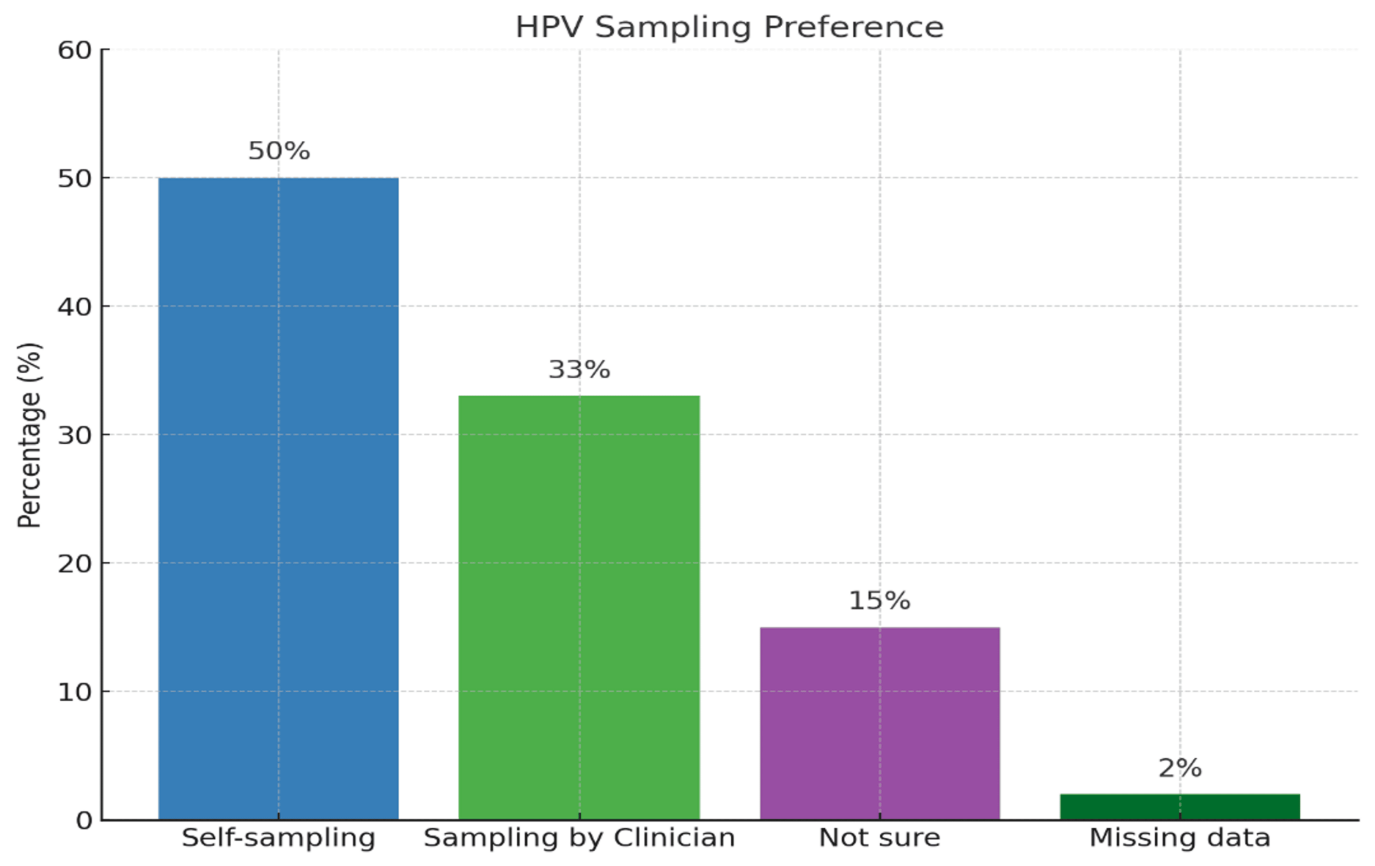
Preference of sampling by study participants Percentage = numbers, as the total participants are 100. HPV: human papillomavirus.

Confidence in performing self-sampling was reported by 97 participants. Among them, 63 (64.9%) felt confident in their ability to self-sample, while 34 (35.0%) expressed uncertainty or a lack of confidence (Table 5). When asked about their preferred sampling method, 50 (50%) participants favored self-sampling, 33 (33%) preferred clinician collection, and 15 (15%) were undecided (Figure 3).

A chi-square test was used to evaluate the association between nationality (UAE versus non-UAE) and sample collection preference (self-collection, clinician-collection, or not sure). The test revealed no significant association between nationality and test preference (χ²(2) = 0.012, p = 0.994). This indicates that both local and non-local participants had similar preferences regarding sample collection method (Table 6).

**Table 6:** HPV sampling preference by nationality of study participants (n, %) Values represent the number and percentage of participants from the UAE/non-UAE group showing sampling preference. A chi-square test was performed to assess the association between nationality and preferred sample collection method. The result was not statistically significant: χ²(2) = 0.012, p = 0.994. The significance threshold was set at p < 0.05. HPV: human papillomavirus.

Nationality	Self-collect n (%)	Clinician-collect n (%)	Not sure n (%)	Missing data n (%)	Total n (%)	Chi-square test (df = 2)	p-value
UAE	36 (49.3%)	24 (32.9%)	11 (15.1%)	2 (2.7%)	73 (73.0%)	χ² (2) = 0.012	0.994
Non-UAE	14 (51.9%)	9 (33.3%)	4 (14.8%)	0 (0.0%)	27 (27.0%)		

## Discussion

HPV self-sampling offers an innovative and empowering approach to cervical cancer screening by enabling women to collect their own specimens privately and conveniently [1]. In line with WHO recommendations, HPV testing is now endorsed as the primary screening method, with five-year intervals, offering a potentially cost-effective enhancement to healthcare systems [5]. Despite its high global acceptability among end users [2], self-sampling was not widely integrated into screening programs until recently, with the Netherlands being the first to adopt high-risk HPV testing with a self-sampling option at the national level [5].

To our knowledge, this is the first study in the United Arab Emirates to evaluate self-sampling using the Qvintip device, although numerous studies have been conducted globally. Attitudes toward self-sampling may vary across regions and demographic groups, warranting further exploration [6]. This study demonstrated substantial concordance between HPV results from self- and clinician-collected samples, supported by a high kappa value indicating agreement beyond chance. Based on Altman's (1991) classification, kappa values are interpreted as follows: poor (<0.2), fair (0.2-0.4), moderate (0.4-0.6), good (0.6-0.8), and very good (0.8-1.0) [7]. High participant acceptability and preference suggest that self-sampling is a feasible, patient-centered strategy for integration into cervical cancer screening programs.

Several studies have demonstrated varying but generally high levels of agreement between self-collected and clinician-collected HPV samples. In a Mexican study, self-sampling showed a higher HPV-DNA prevalence (22.8%) compared to clinician collection (19.2%), with fair agreement (concordance: 92.5%, kappa: 0.40, p < 0.001) [3]. In contrast, a study in Ghana reported excellent agreement (concordance: 94.2%, kappa: 0.88) [8], while a Danish study found comparable prevalence between self- and clinician-collected samples (19.3% versus 18.4%) [9]. Among HIV-positive women in Ethiopia, high-risk HPV prevalence was 29.4% with self-sampling and 23.9% with clinician sampling, with substantial agreement (concordance: 87.3%, kappa: 0.68, p < 0.001) [10]. Meta-analyses further support these findings: one including data from 26 countries reported concordance rates between 87% and 97.5% [11], while another involving 10,071 participants across 26 studies found an overall agreement of 88.7% and a kappa of 0.72, indicating good reliability between methods [12].

The findings of this study indicate that HPV self-sampling was generally well-accepted, with 95% of participants rating the process as easy, 91% as comfortable, 84% reporting no pain, 83% experiencing no embarrassment, and 63% expressing confidence in performing the procedure. Approximately 50% of participants preferred self-sampling over clinician-based collection. These results align with studies conducted globally. For instance, a Hong Kong study reported that 95% found the procedure easy, 86% convenient, and 80% felt confident in performing it, with 69% expressing preference for self-sampling as a screening option [5].

A European study using the Delphi self-lavage and Hybrid Capture (HC) cervical sampler reported that 68% of women preferred self-sampling, describing it as easy (94.3%), painless (94.3%), and not uncomfortable (89.9%) [13]. Similarly, a Mexican study found over 94% of participants found the procedure easy, felt confident performing it, and were willing to repeat it [3]. In China, 42.8% accepted self-sampling, valuing its convenience (94.3%), privacy (91.6%), and lack of embarrassment or pain; however, concerns about accuracy and correct technique were common among those who did not [14]. In rural El Salvador, 68% accepted self-sampling, with 38.8% preferring it for privacy and comfort, while others favored clinician collection due to perceived reliability and provider expertise [15]. Systematic reviews and meta-analysis also support high acceptability across diverse settings and populations, although concerns about collecting an accurate sample contributed to some preferring clinician collection [11, 16, 17].

A recent study in China reported high acceptability of HPV self-sampling, with a mean score of 4.2 out of 5, 33% of participants preferred self-sampling, 27% clinician-sampling, while 40% had no preference. Acceptability was significantly higher among women with better HPV knowledge and prior positive experience with self-sampling (p < 0.05) [18]. A global meta-analysis involving 154 studies and 482,271 women found that self-sampling nearly doubled screening uptake compared to clinician-collected samples, supporting its role in reaching under-screened populations [19].

Also, a systematic review and meta-analysis of randomized controlled trials demonstrated that self-sampling invitations significantly increased participation across all strategies compared to conventional clinician invitations [20]. Improving HPV-related knowledge and public awareness is essential to enhance acceptance and participation in self-sampling programs [14]. In Canada, where primary HPV testing is being introduced, a web-based survey highlighted the importance of understanding public attitudes and beliefs to inform targeted communication strategies aimed at increasing uptake, particularly in inadequately screened groups [21]. While some countries have already integrated self-sampling into national cervical cancer screening programs, others are piloting its implementation, recognizing its potential to overcome key barriers to participation and improve screening adherence [22].

Limitations of study

This pilot study has several limitations. The small sample size may limit the generalizability of the findings to the broader population in the United Arab Emirates. Additionally, the acceptability responses may have been influenced by response bias, as underlying reasons for favorable reporting were not assessed. Despite these constraints, the study offers a basis for promoting cervical cancer screening and increasing awareness of HPV. Given the novelty of self-sampling in the UAE, public education is essential. Future research with a larger, more representative sample is recommended to better understand the uptake and determinants of HPV self-sampling.

## Conclusions

This pilot study demonstrates that HPV self-sampling is a feasible, acceptable, and potentially effective approach for primary screening of cervical cancer in a multicultural UAE population, with a high level of agreement between self- and clinician-collected HPV samples and strong participant satisfaction. However, the small sample size and single-center design may limit the generalizability of the findings. Additionally, possible response bias in acceptability reporting and the lack of data on home-based self-sampling warrant caution in the interpretation of results. Despite these limitations, the study provides valuable preliminary insights into the cultural acceptability and practical utility of self-sampling in a multicultural setting. To validate these findings and support widespread implementation, larger, multicenter studies involving diverse populations across the UAE are essential. Such research will be critical for developing evidence-based policies that expand screening access and enhance cervical cancer prevention efforts nationwide.

## Appendices

Appendix 1 

**Table 7:** Participant Feedback Questionnaire about HPV sampling techniques Participant Feedback Questionnaire of HPV sampling techniques, about easiness, comfortability, pain felt, embarrassment, confidence in performing self-sampling, and the preference of sampling techniques. Adapted from Obiri-Yeboah et al. [8]. This content is licensed under the Creative Commons Attribution 4.0 International License (CC BY 4.0) (http://creativecommons.org/licenses/by/4.0/). Table content has been modified by the author to include three more variables: pain, embarrassment, and confidence in self-sampling.

No	Question	
1	How easy was self-sampling for you?	Very easy
		Easy
		Difficult
		Very difficult
2	How comfortable was the process of self-sampling compared with clinician collection?	Very comfortable
		Comfortable
		Somewhat uncomfortable
		Uncomfortable
3	Did you experience any pain or discomfort during the self-collection?	Yes
		No
		Not sure
4	Did you feel embarrassed to collect self-sampling?	Yes
		No
		Not sure
5	Were you confident in performing the self-sampling technique?	Yes
		No
		Not sure
6	Will you prefer self-sampling or clinician collection in the future?	Self-sampling
		Clinician collect
		Not sure
